# The sensitivity of mechanoelectrical transduction response phase to acoustic overstimulation is calcium-dependent

**DOI:** 10.1007/s00424-023-02883-z

**Published:** 2023-11-21

**Authors:** Pierre Hakizimana

**Affiliations:** Department of Biomedical and Clinical Sciences (BKV), Linköping University, 581 83 Linköping, Sweden

**Keywords:** MET channel, Hair cell response phase, Receptor potential phase, Hair cell extracellular calcium, Hair cell extracellular potassium

## Abstract

The Mechanoelectrical transduction (MET) channels of the mammalian hair cells are essential for converting sound stimuli into electrical signals that enable hearing. However, the impact of acoustic overstimulation, a leading cause of hearing loss, on the MET channel function remains poorly understood. In this study, I investigated the effect of loud sound-induced temporary threshold shift (TTS) on the transduction response phase across a wide range of sound frequencies and amplitudes. The results demonstrated an increase in the transduction response phase following TTS, indicating altered transduction apparatus function. Further investigations involving the reduction of extracellular calcium, a known consequence of TTS, replicated the observed phase changes. Additionally, reduction of potassium entry confirmed the specific role of calcium in regulating the transduction response phase. These findings provide novel insights into the impact of loud sound exposure on hearing impairment at the transduction apparatus level and highlight the critical role of calcium in modulating sound transduction. Considering that over 1 billion teenagers and young adults globally are at risk of hearing loss due to unsafe music listening habits, these results could significantly enhance awareness about the damaging effects of loud sound exposure.

## Introduction

The mechanoelectrical transducer (MET) channels in mammalian hair cells serve as remarkable biological detectors, transforming diverse vibrational mechanical energies produced by sound frequencies and intensities into receptor potentials [2, 3, 10, 13, 19, 25–28, 30, 33, 46]. This mechanism underlies our ability to perceive and interpret auditory stimuli. Despite the intricate nature of the human hearing organ, it remains vulnerable to high-intensity sounds, which are a major cause of hearing loss [11]. The World Health Organization has highlighted hearing loss as a significant health issue, impacting over 400 million individuals worldwide. Furthermore, unaddressed hearing loss imposes a substantial annual financial burden that exceeds USD 980 billion [11].

The implications of hearing loss extend beyond its direct impact on auditory perception. It's often linked with a decrease in overall quality of life [7], increased susceptibility to mental health disorders such as depression [34] and dementia [12, 31], and can lead to learning difficulties [31]. Recent findings bring more urgency to this issue. Over a billion young adults and adolescents are now found to be at an elevated risk of hearing loss due to exposure to unsafe sound levels, notably from music-related activities [12]. This escalating risk underscores the pressing need for comprehensive research into the mechanisms and impacts of noise-induced hearing loss. The urgency is compounded by the fact that almost 27% of the world's countries lack appropriate noise exposure regulations [36].

Central to our auditory perception lies the intricate workings of two components of the hair cell receptor potential—the AC and DC components (reviewed in [13]). In a recent study I conducted, it was confirmed that the AC response is generated by the MET channels, and showed that the DC signal is produced by the basolateral K^+^ channels [21], thus confirming an earlier prediction [32]. Remarkably, both of these electrical potentials can be reliably recorded extracellularly [21]. Additionally, the study revealed an intriguing finding: the DC potential serves as an indicator of auditory health [21]. In normally functioning ears, the DC potential exhibits a positive polarity, but in ears affected by noise-induced impairment, it shifts to negative values [21]. This distinctive shift may provide the brain with a mechanism to differentiate between a weak incoming signal from a healthy ear and one from a damaged ear, potentially influencing subsequent efforts to enhance signal quality [21].

Extensive investigations, covering human subjects and a broad spectrum of animal models, including cats, guinea pigs, mice, pigeons, and chicks, have established the pivotal role of the MET channel AC potentials in encoding sound frequencies and amplitudes [2, 3, 10, 19, 25–27, 27, 30, 33, 46]. This encoding mechanism enables us to discriminate between soft and loud sounds, as well as between dark and bright sounds. In the low-frequency cochlear region, the conversion of AC signals into neural signals, or action potentials, is perfectly phase-locked within a specific time window of the AC signal [37, 42]. The phase of the MET channel AC response is of paramount importance for a range of auditory tasks, including sound localization [20], frequency discrimination, speech perception, and hearing in noisy environments [29, 35].

However, despite the critical role of the AC response phase in hearing, there is a significant knowledge gap regarding how it is affected by TTS-inducing loud sound stimulation.

To address this issue, it becomes necessary to scrutinize the low-frequency AC response phase under different conditions—primarily comparing control scenarios with those following exposure to TTS-inducing loud sounds. Such a comparative examination could shed light on the less understood aspects of our auditory system's vulnerability to high-intensity noise, something the present study undertakes to address.

## Results

The guinea pig temporal bone preparation is a widely used cochlear model for investigating sound transduction mechanisms [5, 14–16, 18, 21–24, 45, 47, 49]. Recently, I employed a meticulous approach using the experimental preparation above to compile a rich dataset of sound transduction AC responses [21]. The dataset encompassed recordings of sound-evoked electrical responses across various conditions, including simulations of TTS, and involved eight sound intensities (ranging from 41 to 71 dB SPL) and 20 frequencies (ranging from 60 to 820 Hz) [21]. The primary focus of this investigation was to characterize the AC and DC amplitudes under these conditions [21]. Notably, the findings revealed that the polarity of the DC serves as a robust indicator of the health condition of the ear, underscoring the significance of this primary outcome [21].

In the present study, the original datasets described above were utilized to investigate the phase of the AC response across various stimulus combinations and conditions. This approach aimed to provide deeper insights into the underlying mechanisms of TTS, as outlined below.

## The AC response phase is normally frequency-sensitive and SPL-resilient

To characterize the sound-evoked AC response phase behaviour across frequencies and stimulus intensities in normal conditions, the sound-evoked AC responses from 20 preparations from 20 different animals under control conditions [21] were subjected to Fourier analysis in MATLAB and the phase data extracted.

**Fig. 1:**
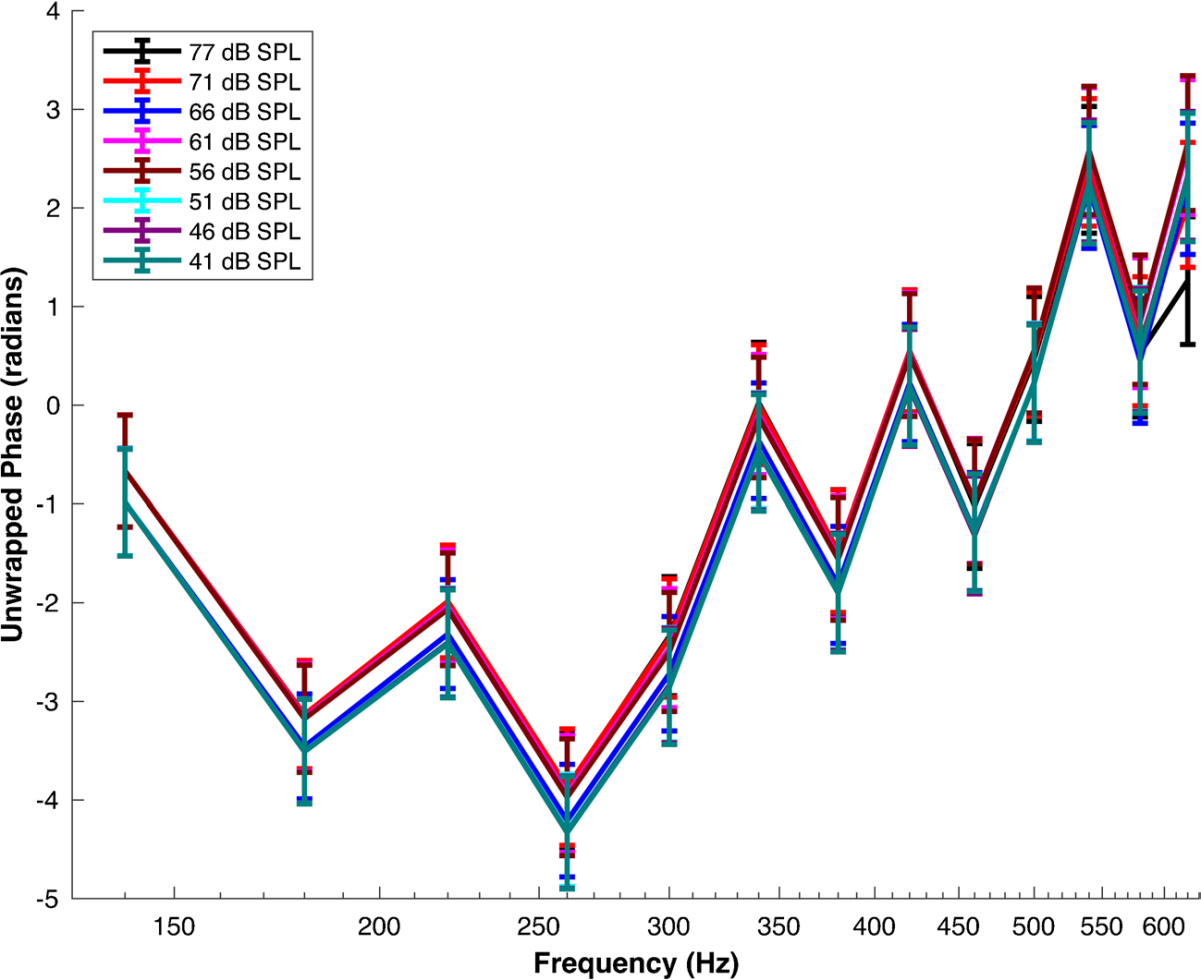
The AC Response Phase Is Normally Frequency-Sensitive and SPL-Resilient. Mean AC response phase versus frequency in control conditions at different sound pressure levels (SPL) of 77, 71, 66, 61, 56, 51, 46, and 41 dB SPL, respectively. The figure labels provide specific color assignments for each experimental condition and SPL combination. The data were obtained from N = 20 preparations sourced from 20 different animals. Error bars indicate the standard error of the means. The corresponding raw waveforms can be viewed in Fig. 2a in Ref [21]

Figure 1 illustrates the AC response phase across the retained frequency range of 140–620 Hz, which was selected based on the observation that the AC amplitude diminishes towards zero outside of this range [21].

As depicted by Fig. 1, the AC response phase exhibits an oscillatory pattern that transitions from predominantly negative phase shifts to positive phase shifts around 340 Hz. A 77 dB SPL for instance, the highest stimulus intensity tested, the phase begins at -0.67 ± 0.57 radians (mean ± s.e.m., n = 20 preparations from 20 different animals) at a frequency of 140 Hz, suggesting an initial phase lag in the AC response. As the frequency increases, the phase delves deeper into the negative territory, reaching its lowest point at -3.89 ± 0.59 radians (n = 20 preparations from 20 different animals) at 260 Hz. Notably, this decline, relative to the data at 140 Hz, proved to be statistically significant (p < 0.0001), as determined by the Wilcoxon signed-rank test.

However, past this frequency point, the phase begins to trend upward and even transitions into positive territory around 420 Hz, denoted by a phase value of 0.54 ± 0.59 radians (n = 20 preparations from 20 different animals). This upward trend suggests a shift from phase lag to phase lead in the AC response. The increase in phase from 260 to 420 Hz was also found to be statistically significant (p < 0.0001), as determined by the Wilcoxon signed-rank test.

The phase continues to oscillate in both positive and negative territories until the end of the frequency range at 620 Hz, with the final phase reading of 1.26 ± 0.65 radians (n = 20 preparations from 20 different animals). When comparing the phase at 620 Hz to that at 420 Hz, the increase was statistically significant (p < 0.001, Wilcoxon signed-rank test). Notably, similar fluctuations in phase have been observed in in vivo recordings of the basilar membrane, as demonstrated in Fig. 1a of ref [17]

Surprisingly, a close examination of Fig. 1 revealed that the oscillatory pattern is consistent across a wide range of sound pressure levels, from 41 to 77 dB SPL. This suggests that the AC response phase is largely independent of the stimulus intensity, which contrast with studies of the basilar membrane displacement phase [17, 43].

To further characterize the effect of sound intensity on the phase, the phase vs frequency functions for different SPL values were subjected to linear mixed modelling (LMM, see methods).

The LMM model was formulated with phase as the dependent variable, with sound pressure level (SPL), group, and frequency as independent variables. The model includes 8 fixed effect coefficients corresponding to distinct SPL levels and 13 random effect coefficients accounting for the variability linked to different frequencies.

Interestingly, the model estimates revealed that the coefficients corresponding to varying SPL levels were relatively small and statistically insignificant (all p-values > 0.05). This suggests that variations in SPL do not significantly influence the phase response, thus confirming the visual trends observed in the data and reinforcing conclusion above that the AC response phase is largely unaffected by sound stimulus intensity.

Furthermore, the random effects for frequency showed a significant standard deviation of 1.90, with a 95% confidence interval ranging from 1.29 to 2.8. This non-zero standard deviation indicates considerable variability in the intercepts across different frequencies. This variability not accounted for by the SPL levels indicates that the phase response is inherently tied to frequency and not to the intensity of the stimulus.

In summary, the absence of significant fixed effects for SPL and the presence of significant random effects for frequency provide robust statistical evidence for the observed oscillatory behaviour of the phase-frequency function across different SPLs. This aligns with the broader observation that the AC response phase is largely invariant to the stimulus intensity.

## Acoustic overstimulation increases the AC response phase across frequencies

To investigate the effect of TTS-inducing acoustic overstimulation, AC response phase data were computed from hair cell electrical responses recorded in 10 preparations from 10 animals after acoustic overstimulation [21].

**Fig. 2:**
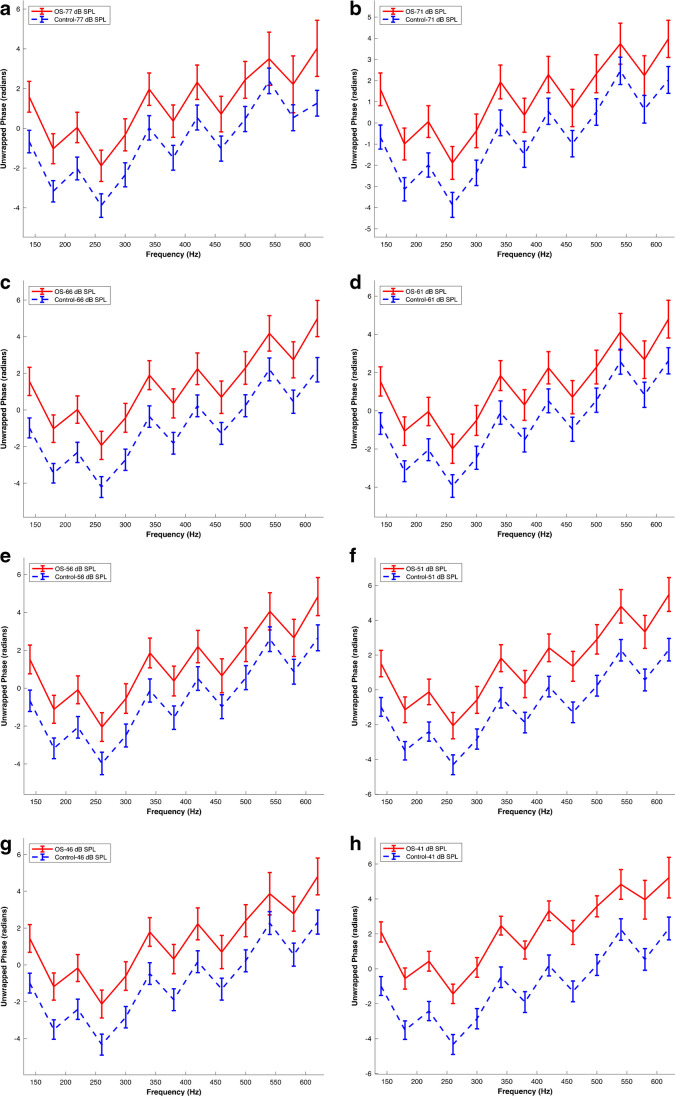
Acoustic Overstimulation Increases The AC Response Phase Across Frequencies. Mean AC response phase versus frequency following acoustic overstimulation. Panels (a) to (h) represent phase data at different sound pressure levels (SPL) of 77, 71, 66, 61, 56, 51, 46, and 41 dB SPL, respectively. Distinctive color-coding for each experimental condition and SPL combination is detailed in the figure labels. The data were obtained from N = 10 preparations sourced from 10 different animals. Error bars indicate the standard error of the means. O.S., acoustic overstimulation. The corresponding raw waveforms can be viewed in Fig. 3a in Ref [21]

Figure 2 presents the post-acoustic overstimulation AC response phases alongside the control data described above for comparison, revealing a noticeable difference in phase responses. The overstimulation dataset consistently exhibited higher phase values compared to the control dataset across all SPLs. At the initial frequency of 140 Hz, the phase values in the overstimulation dataset were consistently higher than those in the control dataset for all SPLs. For instance, at 77 dB SPL, the phase increased from -0.67 ± 0.57 radians (n = 20 preparations from 20 different animals) in the control dataset to 1.59 ± 0.78 radians (n = 10 preparations from 10 different animals) in the overstimulation dataset.

Similarly, at the final frequency of 620 Hz, the phase values in the overstimulation dataset were consistently higher than those in the control dataset for all SPLs. For example, at 77 dB SPL, the phase increased from 1.26 ± 0.65 radians (n = 20 preparations from 20 different animals) in the control dataset to 4.02 ± 1.41 radians (n = 10 preparations from 10 different animals) in the overstimulation dataset.

Importantly, these observed trends were consistent across the entire range of SPLs and frequencies studied (Fig. 2). At each SPL, the phase values in the overstimulation dataset consistently exceeded those in the control dataset, indicating a consistent effect of acoustic overstimulation on phase responses. The similarity of trends across SPLs and frequencies further underscores the consistent impact of acoustic overstimulation on AC response phase values.

This consistency across different SPLs and frequencies suggests a global phenomenon rather than isolated effects at specific SPLs or frequencies. It indicates that acoustic overstimulation has a widespread impact on the resonant behaviour or frequency selectivity of the MET channels, affecting the entire frequency range studied.

To further characterize the effect of TTS on the phase, phase vs frequency functions obtained from the control and acoustic overstimulation datasets were analysed using LMM. The LMM model included fixed effects coefficients for the variables Group (control vs OS), SPL, and an intercept term, while the random effects coefficients accounted for the variability associated with different frequencies.

The fixed effects coefficients revealed important findings. The variable 'Group Acoustic overstimulation' showed a significant increase in phase values for the acoustic overstimulation group compared to the control group (p-value: 1.4 × 10^–144^). However, the coefficients associated with different SPL levels (71 dB SPL, 66 dB SPL, 61 dB SPL, 56 dB SPL, 51 dB SPL, 46 dB SPL, and 41 dB SPL) did not show statistically significant differences in phase values compared to the reference level (p-values > 0.05).

The random effects analysis indicated a significant random effect for the 'Frequency' variable, suggesting variability in intercepts across different frequencies. The estimated standard deviation for 'Frequency' was 2.267, indicating potential differences in phase responses associated with frequency. The random effects covariance parameters also included an estimation of the residual standard deviation, which was 2.8374.

In summary, the statistical analysis confirmed a significant increase in AC phase values following acoustic overstimulation across frequencies, while no significant effects were observed for SPL levels. The presence of a random effect for frequency indicated variability in phase responses across different frequencies.

## TTS-Characterizing extracellular calcium reduction leads to AC response phase increase

Previously, we provided compelling evidence for the role of extracellular calcium in TTS-induced by acoustic overstimulation. We demonstrated that TTS not only leads to a decrease in AC response amplitude [21, 45] but also correlates with a reduction in extracellular calcium levels [45]. This intriguing correlation suggests that the manipulation of extracellular calcium may serve as a potential approach to emulate aspects of TTS. To further support this hypothesis, the MET channel AC response phase was computed in the sound evoked AC responses from 5 different preparations from 5 different animals recorded after the application of a micromolar concentration of ethylene glycol-bis(2-aminoethylether)-N,N,N',N'-tetraacetic acid (EGTA), a calcium chelator known to reduce extracellular calcium concentrations.

**Fig. 3:**
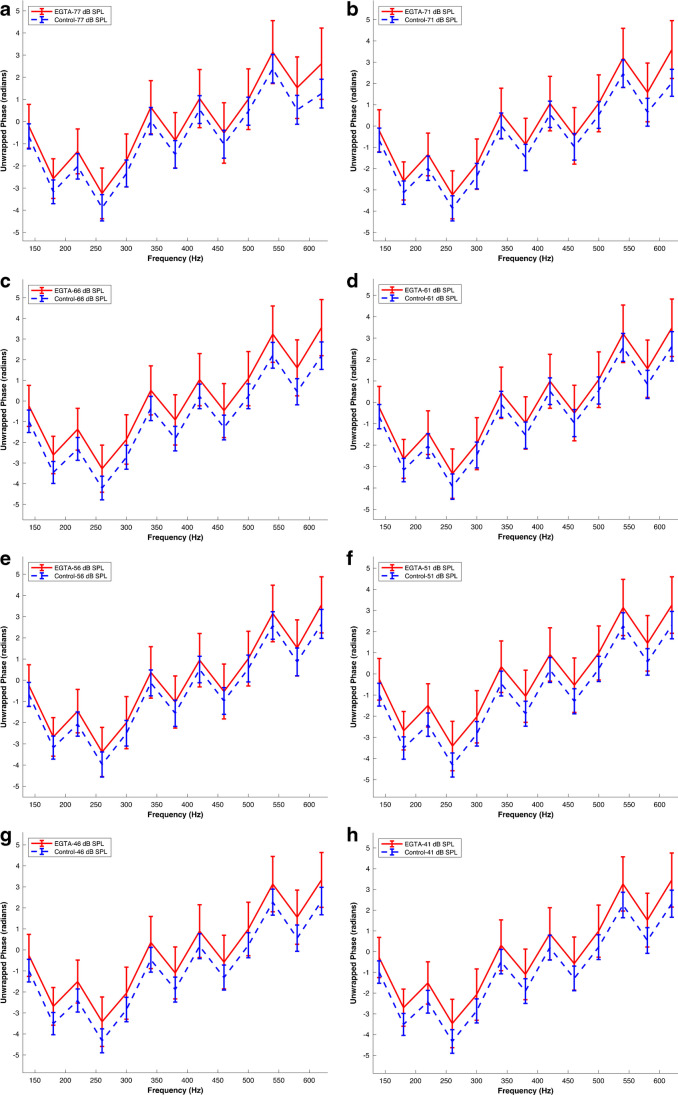
TTS-Characterizing Extracellular Calcium Reduction Leads to AC Response Phase Increase. Mean AC response phase versus frequency following extracellular calcium reduction with micromolar administration of EGTA (see Methods). Panels (a) to (h) represent phase data at different sound pressure levels (SPL) of 77, 71, 66, 61, 56, 51, 46, and 41 dB SPL, respectively. A color key in the figure labels differentiates each experimental condition and SPL combination. The data were obtained from N = 5 preparations sourced from 5 different animals. Error bars indicate the standard error of the means. The corresponding raw waveforms can be viewed in Fig. 5a in Ref [21]

Figure 3 depicts a comparison between the control phase data described above and those obtained after EGTA application, highlighting the distinct differences in phase responses between the two datasets. At the initial frequency of 140 Hz, the AC response phase values in the EGTA dataset consistently exceeded those in the control dataset for all sound pressure levels (SPLs). For instance, at 77 dB SPL, the phase in the control dataset was -0.67 ± 0.57 radians (n = 20 preparations from 20 different animals), while the corresponding value in the EGTA dataset was -0.21 ± 0.99 radians (n = 5 preparations from 5 different animals).

Similarly, at the final frequency of 620 Hz, the phase values in the EGTA dataset exhibited an increase compared to those in the control dataset across all SPLs. For example, at 77 dB SPL, the phase in the control dataset was 1.26 ± 0.65 radians (n = 20 preparations from 20 different animals), while the corresponding value in the EGTA dataset increased to 2.61 ± 1.61 radians (n = 5 preparations from 5 different animals).

Importantly, these observed trends persisted across the entire range of SPLs and frequencies studied, indicating a widespread impact of EGTA treatment on the resonant behaviour or frequency selectivity in the AC response.

To further characterize the effect of EGTA on the AC response phase, the phase vs frequency functions obtained from the control and EGTA datasets were analysed using a linear mixed-effects model (LMM). The fixed effects coefficients provided significant findings, with the 'Group EGTA' variable demonstrating a significant increase in phase values for the EGTA group compared to the control group (p-value: 3 × 10^–16^), with an estimated increase of 0.88 radians. However, in the case of acoustic overstimulation, the coefficients associated with different SPL levels (71 dB SPL, 66 dB SPL, 61 dB SPL, 56 dB SPL, 51 dB SPL, 46 dB SPL, and 41 dB SPL) did not show statistically significant differences in phase values compared to the reference level (p-values > 0.05).

The random effects analysis revealed a significant random effect for the 'Frequency' variable, suggesting variability in intercepts across different frequencies. The estimated standard deviation for 'Frequency' was 2.22, indicating potential differences in phase responses associated with frequency.

In summary, the statistical tests confirmed a significant increase in MET response phases following EGTA treatment, while no significant effects were observed for SPL levels. These results mirror those obtained after acoustic overstimulation and provide further evidence that TTS-induced calcium decrease contributes to an increase in the phase of the AC responses.

## Absence of global MET channel response phase shift following K^+^ entry reduction

The effect of extracellular calcium removal on the AC response amplitude, as identified above, could be attributed to the previously described reduction in AC response amplitude or the specific influence of calcium on the MET channel, as supported by previous studies. To determine the dominant factor between these hypotheses, the AC response phase data were computed from AC recordings from 7 preparations from 7 different animals after treatment with a micromolar concentration of dihydrostreptomycin [21], which specifically reduces the AC response amplitude by reducing K^+^ entry into the MET channels [21, 41].

**Fig. 4:**
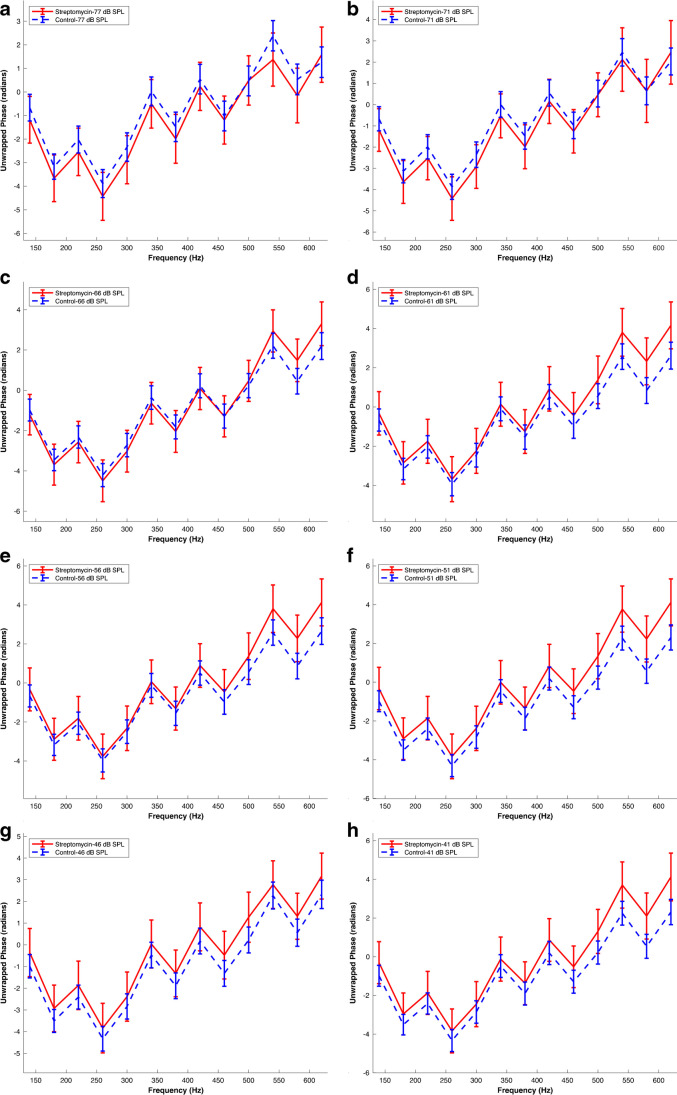
Absence of Global MET channel Response Phase Shift Following K^+^ Entry Reduction. Mean AC response phase versus frequency following micromolar dihydrostreptomycin application (see Methods). Panels (a) to (h) represent phase data at different sound pressure levels (SPL) of 77, 71, 66, 61, 56, 51, 46, and 41 dB SPL, respectively. Each experimental condition and SPL combination is detailed in the figure labels. The data were obtained from N = 7 preparations sourced from 7 different animals. Error bars indicate the standard error of the means. Streptomycin denotes dihydrostreptomycin. The corresponding raw waveforms can be viewed in Fig. 4a in Ref [21]

Figure 4 compares the control phase data with those obtained from 7 preparations after dihydrostreptomycin treatment, revealing a noticeable difference in phase responses across different SPLs and frequencies, with phase lead switching between the control and dihydrostreptomycin datasets at different SPLs. This suggests an alteration in the timing of the auditory response due to dihydrostreptomycin treatment.

At the higher SPLs (77 dB and 71 dB) for both 140 Hz and 620 Hz, the control dataset generally exhibits larger or similar phase values compared to the dihydrostreptomycin dataset, indicating a phase lead or similarity in the control dataset at these SPLs (Fig. 4). However, at lower SPLs (61 dB to 41 dB), the dihydrostreptomycin dataset exhibits larger phase values.

When comparing the trends at 140 Hz and 620 Hz, similar patterns are observed. For instance, at 77 dB SPL, the phase at 140 Hz in the control dataset (-0.67 ± 0.57 radians, n = 20 preparations from 20 different animals) is greater than in the dihydrostreptomycin dataset (-1.18 ± 0.99 radians), while the phase values are closely similar at 620 Hz (Control: 1.26 ± 0.65 radians, n = 20 preparations from 20 different animals; Dihydrostreptomycin: 1.58 ± 1.17 radians, n = 7 preparations from 7 different animals).

At lower SPLs, this trend is reversed. For example, at 61 dB SPL, the phase at 140 Hz in the dihydrostreptomycin dataset (-0.32 ± 1.11 radians, n = 7 preparations from 7 different animals) is larger than in the control dataset (-0.67 ± 0.56 radians, n = 20 preparations from 20 different animals), a trend that also holds at 620 Hz (Dihydrostreptomycin: 4.16 ± 1.2 radians, n = 7 preparations from 7 different animals; Control: 2.62 ± 0.68 radians, n = 20 preparations from 20 different animals).

These changes in phase lead with varying SPLs indicate that the effects of dihydrostreptomycin on the MET channel depend on the loudness of the sound stimulus. The phase lead in the control dataset at higher SPLs suggests a faster auditory response to louder sounds under normal conditions. However, the shift to a phase lead in the dihydrostreptomycin dataset at lower SPLs suggests that dihydrostreptomycin treatment alters this relationship, leading to a quicker response to quieter sounds.

To investigate whether the overall effect of dihydrostreptomycin cancels out across SPLs, LMM was utilized, incorporating an interaction term to account for the apparent interaction between dihydrostreptomycin and SPL.

Notably, the model results indicate that the main effect of the 'Group Dihydrotreptomycin' variable (Estimate: -0.42181, SE: 0.32464, pValue: 0.19) is not statistically significant. This suggests that there is no overall significant difference in phase responses between the control and dihydrostreptomycin groups when not considering different SPLs. This result deviates from the findings observed in control vs. acoustic overstimulation and control vs. EGTA comparisons, where the treatment effect had a significant estimate.

However, the model reveals significant interaction terms of 'Group Dihydrostreptomycin' with several SPL levels, indicating that the effect of dihydrostreptomycin treatment on phase responses significantly varies depending on the SPL level. Furthermore, the model identifies a significant random effect for 'Frequency' with an estimate of 1.98 for the standard deviation, suggesting variability in phase responses across different frequencies.

The absence of a significant global effect of K^+^ reduction on the AC response phase supports that the observed changes in calcium levels affecting the AC response phase discussed above were not solely driven by the reduction in AC response amplitude. Instead, these findings provide compelling evidence for a specific calcium effect on the mechanotransduction apparatus as the underlying mechanism influencing the AC response phase.

## Discussion

In this study, I aimed to investigate the mechanisms underlying temporary threshold shift at the level of the sound transduction apparatus. I examined the effect of acoustic overstimulation on the AC response phase across frequencies and sound intensities. The findings reveal that acoustic overstimulation globally increased the phase across frequencies. Moreover, through subsequent calcium manipulations, it was demonstrated that this overstimulation-induced phase shift was specifically influenced by calcium's effect on the sound transduction apparatus function, rather than being solely attributed to the reduction in response AC amplitude associated with acoustic overstimulation.

Acoustic overstimulation has been extensively studied for its detrimental effects on auditory function [36]. Previous research has shown that exposure to high sound intensities can lead to temporary or permanent changes in hearing sensitivity [36].

Although this study represents the first investigation of TTS specifically focused on the AC response phase, previous TTS studies have predominantly examined the phase of the basilar membrane and neural response. However, the findings from these studies have been inconsistent, with some reporting a decrease in phase following overstimulation along with an increase in phase with sound pressure levels (SPLs) [17, 43], while others have shown the opposite trend [38, 40]. Notably, one study observed no significant effect of SPL on the phase up to 75 dB SPL under control conditions [38], which is consistent with the present study's findings. However, it should be noted that the phase data from the basilar membrane may not necessarily correspond directly to the neural phase, especially at higher SPLs [44], suggesting that the findings from one cochlear structure may not be directly transferable to another.

The present study revealed a notable increase in phase across frequencies following exposure to acoustic overstimulation, which could disrupt the precise timing of the auditory response. Importantly, the identification of a specific effect of calcium on the function of the sound transduction apparatus in this study suggests a mechanistic connection between calcium dynamics and the observed changes in phase responses during TTS and agrees with past studies that have linked calcium to hearing sensitivity [1, 6, 8, 39].

Understanding the effect of acoustic overstimulation on the phase responses is crucial for unravelling the complex mechanisms underlying TTS. By examining the role of the sound transduction apparatus and calcium dynamics in modulating the phase response, the present study provides valuable insights into the specific factors contributing to altered temporal processing following overstimulation.

## Materials and methods

The sound-evoked electrical recordings and the experimental procedures associated with data acquisition have been extensively described in the primary study [21].

## The guinea pig temporal bone preparation and electrophysiological recordings

The methodologies employed were approved by the Regional Ethics Committee in Linköping, Sweden (Approval DNR 5111–2019). Guinea pigs, regardless of gender and aged 2–5 weeks, were anesthetized using sodium pentobarbital and subsequently prepared for the procedure. Subsequently, the left temporal bone was isolated and mounted onto a rotative stand, an integral part of a custom-made chamber. Immediately, the bulla was gently opened to expose the cochlear base and apex. The preparation was then promptly submerged in an oxygen-rich cell culture medium. Incisions were swiftly made at both the base and apex, with the apical opening used for detailed measurements in this study.

A continuous flow of the same oxygen-rich medium was established through the cochlea via the basal opening, using thin tubing sourced from an external 4 ml-tank. This perfusion system kept the preparation vital for up to four hours [4]. Specific steps of this procedure have been extensively detailed in other publications [21, 23, 45, 47]. Owing to the swift setup, typically finalized within an hour post-decapitation, ample time was provided for experiments under consistent conditions. The custom chamber's design ensured that the ear canal remained free from fluids, allowing the precise delivery of sound stimuli via a calibrated speaker. A notable consideration is that middle ear fluid immersion can diminish the sound stimulus by at least 20 dB SPL [23, 48]. Thus, all sound intensities cited in this study have been adjusted accordingly. The chamber's design permitted secure attachment to the microscope stage using a screw system. Furthermore, the rotative stand enabled the apical opening's tilt, facilitating smooth penetration of the recording glass electrode into the reissner’s membrane, close and parallel to the stria vascularis. This was achieved seamlessly with a computer-controlled step motor under the guidance of reflected light confocal imaging (see Fig. 1 in ref [21] for the electrode penetration site). To enhance precision and minimize external interferences, both the microscope and preparation were safely housed within a light-shielded Faraday chamber and placed atop a vibration-resistant table.

For the sound-evoked response recordings, the glass electrode was filled with an artificial endolymph solution, beveled to 3 megaOhms, and connected to a Dagan Ix1 amplifier. The presentation of stimuli and recording of evoked electrical responses were controlled by custom LabView software [45]. Data digitization was performed by a high-resolution 24-bit A/D board (NI USB-4431; National Instruments) at a 10 kHz frequency. When necessary, prior to bevelling, the electrode solution was supplemented with either EGTA (100 μM) or dihydrostreptomycin (DHS, up to 100 μM). Drug delivery to the endolymphatic space, housing the stereocilia, was achieved using a Picospritzer by briefly applying a low pressure (≤ 4 psi) at the electrode's rear. This method ensured precise drug application since the dispensed volume was restricted by the designated pressure and the minuscule opening of the bevelled electrode. Drug release ensued exclusively upon pressure engagement, typically resulting in a single small droplet emission from the electrode. Therefore, following delivery, the drug concentration within the endolymphatic space was significantly reduced compared to that in the electrode, since the droplet diffuses away from the injection site (see Fig. 1 in ref [21]) in the direction of the stereocilia. Given that recent studies have demonstrated the specific effect of 0.2 mM dihydrostreptomycin on hair cell MET currents [9], the minimal doses of dihydrostreptomycin employed in this research are unlikely to produce any side effects. As demonstrated in Ref [21], preparations treated with dihydrostreptomycin retained approximately 60% of the control AC response amplitude, underscoring the specificity of dihydrostreptomycin at the administered dose.

The acquisition software [45] was used continually monitor the stability of the sound-evoked electrical responses. After initial recordings, subsequent data acquisitions were color-coded and superimposed, enabling researchers to track the stability of AC and SP tuning curves over time. Once stability was verified for at least 8–15 min, detailed investigations were undertaken, recording responses across 20 distinct frequencies and eight sound intensities, spanning 41 to 77 dB SPL. This data was reserved for subsequent offline analysis using MATLAB. In this investigation, the recordings were analyzed via Fourier analysis in MATLAB, extracting phase information across various SPLs and frequencies. The derived phase data were preserved for ensuing statistical evaluations, quantifications, and graphical representations using MATLAB.

Where indicated, acoustic overstimulation was deployed using a 98 dB SPL at a 140 Hz frequency, slightly below the optimum frequency for the recording localeas previously described [45], with the exposure duration limited to roughly one minute.

## Statistical analysis

In this study, linear mixed modelling (LMM) was conducted using the built-in fitlme function in MATLAB, where indicated. As previously mentioned [21], the measurements of sound-evoked responses were obtained repeatedly at different frequencies within each experimental preparation. This repetition could potentially introduce correlations that needed to be addressed using LMM, as described before [21]. Where indicated, Wilcoxon signed-rank test was performed in MATLAB.

## Data Availability

As stated for the primary study^[[21]]^, all data supporting the findings are available from the corresponding author upon reasonable request.
